# 

**Published:** 1845-09

**Authors:** 


### AMERICAN JOURNAL

OF

### Dental Science.

PUBLISHED UNDER THE AUSPICES OF THE

American Society of Dental Surgeons.

VOLUME VI.—1845-6.

EDITORS:

CHAPIN A. HARRIS M. D., D. D. S.

EDWARD MAYNARD, M. D., D. D. S.

AMOS WESTCOTT, M. D., D. D. S.

BALTIMORE:

ARMSTRONG & BERRY.

JOHN W. WOODS, PRINTER.

1845.

*Washington City Editor.*—Dr. Maynard, the Washington City Editor, is now in Europe, and will, from time to time, during his absence, furnish the readers of the Journal with such information as may be of interest to the members of the profession in this country.

*To Subscribers in Great Britain.*—We have just received the London Forceps, up to No. 31, and in looking over them hastily, we noticed, in No. 23, for November 15th, 1844, a note from one of our London subscribers, headed "The American Journal," in which he asks, "is it likely I shall receive the last two numbers of the 'American Journal of Dental Science,' the first of which was due in June, the second in September." We regret very much, that any thing should prevent our subscribers in Great Britain from receiving their numbers regularly, as we feel under many obligations to them, for the liberal manner in which they have contributed to sustain this publication. We send the numbers regularly, as soon as issued, and have endeavored to make arrangements to have them delivered immediately on their arrival. Hereafter, we hope this will be done, and that none of our transatlantic readers will again have occasion to complain on account of want of punctuality on our part.

*Dental Infirmary.*—It will be seen by the advertisement of the Baltimore College of Dental Surgery, that a dental infirmary is soon to be established, in connection with this Institution.

*To Correspondents.*—The communication from Dr. Elliott shall appear in the next number of the Journal. Dr. T. H. will find an answer to his inquiry, in the proceedings of the American Society of Dental Surgeons.

### EDITORS' PROSPECTUS

OF THE

### American Journal and Library of Dental Science.

As this Journal has become the organ of the American Society of Dental Surgeons, and as its name has been somewhat modified, it may be considered as having entered upon a new period of its existence; and its former publishers congratulate themselves and the profession on its good fortune. Its perpetuity is now considered as settled. The profession will always need a periodical devoted to its interests, and who is better able to conduct it, than the only regularly organized and extensive association of dental surgeons in the world. We repeat, that we congratulate ourselves and our professional brethren on an event so auspicious to the science of dental surgery, as the formation of a national society and the transfer of the journal to that body. The work can now be regularly issued, whether its circulation be co-extensive with the profession or not. If the subscription should be limited, the issues can be made to correspond with the demand, so that the expense to the society may be kept wholly within its means.

The American Journal and Library of Dental Science, is now proposed to take an independent ground, and will hereafter solicit no favours from those members of the profession who are opposed to the dissemination of periodical information. To others who are favourably inclined to a community of knowledge, this journal will not fail to commend itself, as one of the most efficacious means of exposing the impositions of quackery, and imparting to honorable pretensions that kind of information which their usefulness and talents should secure.

The journal will be regularly issued on the first of December, March, June and September, annually, at the enhanced price of five dollars per volume, payable in all cases in advance. The price of subscription may be paid to the treasurer of the society, Dr. C. A. Harris, Baltimore city, or to either of the editors, or any of the publishers, collaborators or acknowledged agents of the journal. Advance payment is required by a special resolution, passed at the last meeting of the society, and is necessary to protect it from pecuniary loss, a neglect of which has involved the publishers of the first volume in unexpected embarrassment, if not in ultimate loss.

To save postage, the agents in all the large cities of the United States, and Great Britain, will be furnished with copies to supply subscribers, by such conveyance as they shall prescribe. And where it can be done, even individuals may receive their numbers by private conveyance if they choose.

With these few explanatory remarks, we commit our work to its coming destiny, after assuring the profession that no efforts will be spared to render it every way worthy of the great subject to which its pages will be exclusively devoted.

CHAPIN A. HARRIS,

EDWARD MAYNARD,

AMOS WESTCOTT.

---

Besides the Collaborators, the following gentlemen are regularly authorized

### AGENTS OF THIS JOURNAL.

Bradbury & Soden,........Boston.

John M'Connell, M. D.....Richmond, Va.

O. P. Laird, M. D.........Columbus, Ga.

Geo. Washington Parmly, New Orleans.

G. McDonald, M. D .......Macon, Ga.

Blandin & Reynolds,.....Columbia, S. C.

Eleazar Gidney, D. D. S...Manchester, Eng.

Drs. Goddard and Ferguson, Louisville, Ky.

James Taylor, M. D.. ....Cincinnati, Ohio.

Dr. A. Vancamp,...........Nashville, Tenn.

C. O. Cone, D. D. S..........Georgetown, Ky.

Nicholas Clute,..... ....Louisville,

John Lees, (now in England,) Philadelphia.

E. J. Dunning,.............Buffalo, N. Y.

Benj. Strickland,.........Cleveland, Ohio.

B. B. Brown, M. D.........St. Louis, Mo.

W. R. Scott, D. D. S........Raleigh, N. C.

S. M. Shepherd,............Petersburg, Va.

John A. Cleveland,.......Charleston, S. C.

Edward Taylor, M. D.. ...Natchez, Miss.

### Acknowledgments of the receipts of subscriptions to the Journal.

| | | | | | |
|---|---|---|---|---|---|
| J. A. Cleaveland, | Vol. 5 | $ 5 00 | T. A. Sale, | Vol. 6 | 5 00 |
| T. P. Cleaveland, | " 5 | 5 00 | L. A Swift, | " 6 | 5 00 |
| James Taylor, | " 6 | 5 00 | J. D. Wimple, | " 6 | 5 00 |
| Edward Taylor, | " 6 | 5 00 | W. R. Scott, | " 6 | 5 00 |
| W. B. Ross, | " 5 | 5 00 | Jedediah Smith, | " 6 | 5 00 |
| A. Berry, | " 5 | 5 00 | C. C. Porter, | " 6 | 5 00 |
| James Clark, | " 4 | 5 00 | N. S. Tyler, | " 5 | 5 00 |
| J. W. Smith, | " 4 & 5 | 10 00 | J. M. Peak, | " 5 | 5 00 |
| H. H. Young, | " 4 & 5 | 10 00 | H. F. Smith, | " 5 | 5 00 |
| Robert Nelson, | " 4 & 5 | 10 00 | John Rees, | " 5 | 5 00 |
| Charles Walker, | " 5 | 5 00 | W. N. Bispham, | " 5 | 5 00 |
| P. Houston, | " 4 & 5 | 10 00 | D. J. Fogle, | " 5 | 5 00 |
| J. M. Howe, | " 4 & 5 | 10 00 | T. B. Hamlin, | " 5 | 5 00 |
| Charles Merritt, | " 3 | 5 00 | James Alcock, | " 5 | 5 00 |
| E. Parmly, | " 6 | 5 00 | J. T. Thornton, | " 5 | 5 00 |
| E. J. Dunning, | " 5 & 6 | 10 00 | David Morrow, | " 3 & 4 | 10 00 |
| H. McCollum, | " 4 | 5 00 | W. G. Ballard, | " 5 | 5 00 |
| J. Fogle, | " 6 | 5 00 | J. P. Dinsmore, | " 4 & 5 | 10 00 |
| Prof. Moncur, | " 4 & 5 | 10 00 | A. B. Hay, | " 5 | 5 00 |
| J. F. Cassell, | " 6 | 5 00 | W. H. Howett, | " 5 | 5 00 |
| J. Taft, | " 6 | 5 00 | Orson Whiting, | " 5 | 5 00 |
| W. O. Laird, | " 5 & 6 | 10 00 | C. C. Allen, | " 4 & 5 | 10 00 |
| T. B. Hamlin, | " 6 | 5 00 | Dr. Rouell, | " 4 & 5 | 10 00 |
| G. Merryman, | " 5 | 5 00 | Dr. Hawes, | " 5 | 5 00 |
| H. W. Tilyard, | " 4 & 5 | 10 00 | F. Crocker, | " 5 | 5 00 |
| O. Holmes, | " 4 & 5 | 10 00 | A. L. Bardwell, | " 6 | 5 00 |
| Andr. Ruddock, | " 4 | 5 00 | Dr. H. Burr, | " 5 | 5 00 |
| Flaig, | " 4 | 5 00 | E. Townsend, | " 5 | 5 00 |
| J. H. Briscoe, | " 4 | 5 00 | Dr. N. S. M'Ilhenny, | " 5 | 5 00 |
| Mr. Ash, | " 4 | 5 00 | F. Reinstein, | " 5 | 5 00 |
| E. Vanderslice, | " 4 | 5 00 | C. C. Williams, | " 5 | 5 00 |
| E. Tracy, | " 4 | 5 00 | S. T. Beale, | " 5 | 5 00 |
| N. L. Dickey, | " 4 | 5 00 | J. M. Harris, | " 5 | 5 00 |
| G. Naglee, | " 3 | 5 00 | J. D. More, | " 5 | 5 00 |
| J. H. Foster, | " 4 | 5 00 | J. W. Lawrence, | " 5 | 5 00 |
| Mr. Carroll, | " 4 | 5 00 | J. E. Parker, | " 5 | 5 00 |
| Dr. Ely Parry, | " 3, 4 & 5 | 15 00 | S. Dillingham, | " 5 | 5 00 |
| C. Walton, | " 5 & 6 | 10 00 | Dr. G. Planton, | " 5 & 6 | 10 00 |
| C. Abbey, | " 5 & 6 | 10 00 | Peter Ebert, | " 5 & 6 | 10 00 |
| J. F. Caldwell, | " 5 | 5 00 | H. P. Montgomery, | " 6 | 5 00 |
| W. W. Fouche, | " 5 & 6 | 15 00 | D. M. C. White, | " 6 | 5 00 |

Not having the names of all the subscribers who have paid their subscriptions to the Journal to Agents, the Treasurer is compelled to omit such. If any others are left out of the foregoing list, the correction will be made on receiving information of the fact.

C. A. HARRIS,

*Treasurer, A. S. D. S.*

### EDITORS' PROSPECTUS

OF THE

### American Journal and Library of Dental Science.

---

As this Journal has become the organ of the American Society of Dental Surgeons, and as its name has been somewhat modified, it may be considered as having entered upon a new period of its existence; and its former publishers congratulate themselves and the profession on its good fortune. Its perpetuity is now considered as settled. The profession will always need a periodical devoted to its interests, and who is better able to conduct it, than the only regularly organized and extensive association of dental surgeons in the world. We repeat, that we congratulate ourselves and our professional brethren on an event so auspicious to the science of dental surgery, as the formation of a national society and the transfer of the journal to that body. The work can now be regularly issued, whether its circulation be co-extensive with the profession or not. If the subscription should be limited, the issues can be made to correspond with the demand, so that the expense to the society may be kept wholly within its means.

The American Journal and Library of Dental Science, is now proposed to take an independent ground, and will hereafter solicit no favours from those members of the profession who are opposed to the dissemination of periodical information. To others who are favourably inclined to a community of knowledge, this journal will not fail to commend itself, as one of the most efficacious means of exposing the impositions of quackery, and imparting to honorable pretensions that kind of information which their usefulness and talents should secure.

To save postage, the agents in all the large cities of the United States, and Great Britain, will be furnished with copies to supply subscribers, by such conveyance as they shall prescribe. And where it can be done, even individuals may receive their numbers by private conveyance if they choose.

With these few explanatory remarks, we commit our work to its coming destiny, after assuring the profession that no efforts will be spared to render it every way worthy of the great subject to which its pages will be exclusively devoted.

CHAPIN A. HARRIS,

EDWARD MAYNARD,

AMOS WESTCOTT.

---

Besides the Collaborators, the following gentlemen are regularly authorized

### AGENTS OF THIS JOURNAL.

Saxton & Ketts,..........Boston.

John M'Connell, M. D.....Richmond, Va.

O. P. Laird, M. D.........Columbus, Ga.

L. S. Parmly, M. D.......New Orleans.

G. McDonald, M. D .......Macon, Ga.

Drs. Goddard and Ferguson,Louisville, Ky.

James Taylor, M. D.. .....Cincinnati, Ohio.

Dr. A. Vancamp,...........Nashville, Tenn.

C. O. Cone, D. D. S..........Georgetown, Ky.

Nicholas Clute,..... ....Louisville,

John Lees, (now in England,) Philadelphia.

Benj. Strickland,.........Cleveland, Ohio.

B. B. Brown, M. D.........St. Louis, Mo.

W. R. Scott, D. D. S.........Raleigh, N. C.

S. M. Shepherd,.............Petersburg, Va.

John A. Cleveland,.......Charleston, S. C

